# H3K27ac‐activated LINC00519 promotes lung squamous cell carcinoma progression by targeting miR‐450b‐5p/miR‐515‐5p/YAP1 axis

**DOI:** 10.1111/cpr.12797

**Published:** 2020-04-16

**Authors:** Peng Ye, Xiayi Lv, Rusidanmu Aizemaiti, Jun Cheng, Pinghui Xia, Meng Di

**Affiliations:** ^1^ Department of Thoracic Surgery The First Affiliated Hospital of Zhejiang University Hangzhou China

**Keywords:** ceRNA, histone acetylation, LINC00519, lung squamous cell carcinoma

## Abstract

**Objectives:**

Long non‐coding RNAs (lncRNAs) are extensively reported as participants in the biological process of diverse malignancies, including lung squamous cell carcinoma (LUSC). Long intergenic non‐protein coding RNA 519 (LINC00519) is identified as a novel lncRNA which has not yet been studied in cancers.

**Materials and Methods:**

LINC00519 expression was detected by qRT‐PCR. The effect of LINC00519 on LUSC cellular activities was determined by in vitro and in vivo assays. Subcellular fractionation and FISH assays were conducted to identify the localization of LINC00519. The interaction between miR‐450b‐5p/miR‐515‐5p and LINC00519/YAP1 was verified by RIP, RNA pull‐down and luciferase reporter assays.

**Results:**

Elevated level of LINC00519 was identified in LUSC tissues and cell lines. High LINC00519 level predicted unsatisfactory prognosis. Then, loss‐of‐function assays suggested the inhibitive role of silenced LINC00519 in cell proliferation, migration, invasion and tumour growth and promoting effect on cell apoptosis in LUSC. Mechanically, LINC00519 was activated by H3K27 acetylation (H3K27ac). Moreover, LINC00519 sponged miR‐450b‐5p and miR‐515‐5p to up‐regulate Yes1 associated transcriptional regulator (YAP1). Additionally, miR‐450b‐5p and miR‐515‐5p elicited anti‐carcinogenic effects in LUSC. Finally, rescue assays validated the effect of LINC00519‐miR‐450b‐5p‐miR‐515‐5p‐YAP1 axis in LUSC.

**Conclusions:**

H3K27ac‐activated LINC00519 acts as a competing endogenous RNA (ceRNA) to promote LUSC progression by targeting miR‐450b‐5p/miR‐515‐5p/YAP1 axis.

## 1 INTRODUCTION

Lung cancer is a common malignant tumour in the world and causes numerous deaths. As estimated, about 1.6 million people died of lung cancer, which is about 20% of global cancer mortality annually.^1^, ^2^ Nearly 90% of lung cancer patients are diagnosed with non‐small cell lung cancers (NSCLC), and activation of various oncogenes is confirmed to be vital for NSCLC initiation.^3^, ^4^ LUSC is a subtype of NSCLC, and its molecular characterization has not been sufficiently explained in the past decades. However, LUSC is responsible for the increasing incidence and deaths each year globally.^5^ Although great progress has been made in the therapeutic strategies, the prognosis of LUSC patients is still unfavourable, especially in advanced patients.^5^ Therefore, it is quite urgent to elucidate the mechanism and search the effective biomarkers for LUSC patients.

Through many years of research, a large portion of human non‐coding genome is revealed due to the development of large‐scale sequencing. The lncRNAs are new‐founded members which occupies a large portion of ncRNA family. Their length is over 200 nucleotides and they have limited or no capacity of coding proteins. Abnormally expressed lncRNAs have been identified in hepatocellular carcinoma,^6^ cervical cancer,^7^ gastric cancer^8^ and pancreatic cancer,^9^ suggesting that the dysregulation of certain lncRNAs contributes to tumorigenesis. In recent years, lncRNAs have been commonly acknowledged to affect a variety of biological processes, such as regulating proliferation and apoptosis and reprogramming the pluripotency of stem cells. As recently reported, lncRNA can act as a tumour facilitator or suppressor in cancers.^10^, ^11^ These results revealed that lncRNAs exhibited pivotal functions in regulating genome.

In terms of mechanism, lncRNAs can impact the function of transcriptional complexes and modulate chromatin structures via acting as scaffolds between proteins or serving as the sponges of microRNA.^12^, ^13^ Researches during the past decade have improved the characterization of the function and mechanism of lncRNAs, but thorough understanding of lncRNA‐related mechanism in cancer remains insufficient.

A growing body of reports have established the correlation between lncRNAs and LUSC. For example, CASC9 is up‐regulated in LUSC tissues and plays a promoted role in the proliferation of LUSC cells.^14^ SNHG1 is reported as an oncogene in LUSC that promotes LUSC cell invasion and metastasis.^15^ Our study discovered LINC00519 as a novel up‐regulated lncRNA in LUSC based on bioinformatics databases, and LINC00519 has not been studied in cancers yet. Therefore, we planned to characterize the role and mechanism of LINC00519 in LUSC.

## 2 MATERIALS AND METHODS

### 2.1 Cell lines and reagent

LUSC cells (H266, SK‐MES‐1) and human normal bronchial epithelial cell (HBE) were both procured from Chinese Academy of Sciences and maintained in DMEM (Gibco) at 37°C supplied with 5% CO_2_ atmosphere. 10% foetal bovine serum (FBS) from Gibco was utilized to supplement DMEM. Besides, 10 μmol/L of C646, the histone acetyltransferase (HAT) inhibitor, was bought from Selleck Chemicals. 5 µmol/L of XMU‐MP‐1 (Hippo pathway inhibitor) and DMSO were both obtained from Sigma‐Aldrich.

### 2.2 Tissue specimens

Fifty pairs of LUSC and matched adjacent tissue samples were collected from recruited LUSC patients between 2013 and 2018, with the signed informed consents from all participants and the approval from the Ethical Review Board of the First Affiliated Hospital of Zhejiang University. None of patients was subjected to radiotherapy or chemotherapy prior to operation. All samples were instantly preserved in liquid nitrogen at −80°C for further use.

### 2.3 qRT‐PCR

TRIzol reagent was obtained commercially from Invitrogen for extracting total cellular RNAs from H266 and SK‐MES‐1 cells. PrimeScript RT reagent kit was produced by Takara Bio for reverse‐transcription from total RNA to cDNA. SYBR Green PCR Master Mix (Takara) was employed for quantitative PCR on StepOnePlus System (Applied Biosystems). Fold change of gene level was determined by $2^{-\Delta \Delta C_{T}}$ method, with U6 or GAPDH as normalization. Primer sequences are as follows:

LINC00519

F: 5′‐TGGAGACTTGCTCATGCCAG‐3′
R: 5′‐TGCAACGCAGACAAAATGGG‐3′

CBP

F: 5′‐AACGGGTGAGTGAACTGCTT‐3′
R: 5′‐GCGTTTGGCCAGCAAACTAA‐3′

P300

F: 5′‐CAATGAGATCCAAGGGGAGA‐3′
R: 5′‐ATGCATCTTTCTTCCGCACT‐3′

YAP1

F: 5′‐TAGCCCTGCGTAGCCAGTTA‐3′
R: 5′‐TCATGCTTAGTCCACTGTCTGT‐3′

GAPDH

F: 5′‐TTTAACTCTGGTAAAGTGGA‐3′
R: 5′‐GAATCATATTGGAACATGTA‐3′

MiR‐450b‐5p

F: 5′‐GCTTTTGCAATATGTTCCG‐3′
R: 5′‐CAGTGCGTGTCGTGGAGT‐3′

MiR‐515‐5p

F: 5′‐CGGGTTCTCCAAAAGAAAGCA‐3′
R: 5′‐CAGCCACAAAAGAGCACAAT‐3′

U6

F: 5′‐CTCGCTTCGGCAGCACA‐3′
R: 5′‐AACGCTTCACGAATTTGCGT‐3′

### 2.4 Transfection

Transfection kit Lipofectamine 2000 from Invitrogen was utilized in LUSC cells for 48 hours. For gene silencing, the shRNAs specific to LINC00519, CBP (sh‐LINC00519#1:5′‐CCGGGGTACGAACTTTCGATTATAGCTCGAGCTATAATCGAAAGTTCGTACCTTTTTG‐3′; sh‐LINC00519#2:5′‐CCGGGCACCAACCGGGTATTCTACACTCGAG TGTAGAATACCCGGTTGGTGCTTTTTG‐3′; sh‐CBP: 5′‐CCGGCCCGATAACTTTGTGATGTTTCTCGAGAAACATCACAAAGTTATCGGGTTTTT‐3′) and control (sh‐NC) were used. The miR‐450b‐5p inhibitor, miR‐515‐5p inhibitor and control (NC inhibitor) were procured from RiboBio to knock down miR‐450b‐5p and miR‐515‐5p. For gene overexpression, the pcDNA3.1/LINC00519, pcDNA3.1/YAP1 and control (empty vector), miR‐450b‐5p mimics, miR‐515‐5p mimics and control (NC mimics), were synthesized by Genepharma.

### 2.5 Cell counting kit‐8

After seeding LUSC cells into the 96‐well plate for 24‐hour incubation, each well was added with 10 μL cell counting kit‐8 (CCK‐8) for cell incubation for 2 hours at 37℃ in the dark. Thereafter, value of absorbance was measured at 450 nm utilizing a microplate detector (EnSpire 2300; PerkinElmer).

### 2.6 Colony formation

Lung squamous cell carcinoma cells transfected for 48 hours were planted to 6‐well plates (5 × 10^3^ cells/well) in an incubator at 37°C with 5% CO_2_. 14 days later, 0.1% crystal violet solution was added after fixation by 5% paraformaldehyde, and colonies were finally counted and recorded.

### 2.7 Flow cytometry of apoptosis

Lung squamous cell carcinoma cells in 6‐well plates were rinsed in phosphate buffer saline (PBS), and then were trypsinized and re‐suspended in 100 μL of binding buffer added with 2.5 μL of fluorescein isothiocyanate (FITC)‐conjugated Annexin V and 1 μL of PI (Invitrogen). Fifteen minutes later, flow cytometry (BD Biosciences) was utilized for apoptotic LUSC cells.

### 2.8 TUNEL assay

Lung squamous cell carcinoma cells were treated with dUTP‐end labelling solution (Clontech), after treatment with 1% formaldehyde and 0.2% Triton X‐100. DAPI solution from Beyotime was added to counterstained cell nuclei, following observation of fluorescence microscope (NIKON).

### 2.9 Wound healing assay

Lung squamous cell carcinoma cells were cultured in 96‐well plates (5 × 10^4^ cells/well) all night to allow cells to adhere, and then, wounds were scratched with sterile pipette tip. Following washing in PBS, wounds were imaged after 24 hours.

### 2.10 Cell invasion assay

Transfected LUSC cells were reaped and seeded to the upper chamber of transwell inserts (8 μm pores; Corning Incorporated) coating Matrigel (BD Biosciences) in 24‐well plates. Invasive cells were cultured with crystal violet and counted with microscope (magnification, ×200).

### 2.11 In vivo experiment

This animal study was approved and performed strictly in accordance with the Institutional Guidelines on the Use of Live Animals in the First Affiliated Hospital of Zhejiang University. Six‐week‐old pathogen‐free male BALB/c‐nude mice were bought from Vital River and subcutaneously injected with transfected LUSC cells. Nude mice were killed after 28 days of injection, and excised tumours were weighed for subsequent analysis.

For metastasis assay, immunodeficiency mice were injected intravenously with transfected LUSC cells. At week 7, all mice derived metastasis tumours and lungs from all mice were collected and fixed in 10% formalin. Then, metastasis was detected with the staining of pathological sections from paraffin‐embedded tissues by standard HE procedure.

### 2.12 Immunohistochemical assay

The tissue samples from in vivo experiment were incubated with 4% paraformaldehyde and then embedded in paraffin. The samples were cut into 4‐μm‐thick sections and assayed by immunohistochemical (IHC) employing the antibodies against Ki‐67 and PCNA (Santa Cruz Biotechnology, Dallas, TX, USA).

### 2.13 Chromatin immunoprecipitation

EZ‑Magna chromatin immunoprecipitation (ChIP) kit was obtained from Millipore for ChIP assay. Cells were cultivated in formaldehyde at room temperature for 10 minutes to acquire DNA‑protein cross‑links. Lysates were sonicated and immunoprecipitated for 1 hour at room temperature with anti‐H3K27ac, anti‐CBP anti‐P300 or control anti‐IgG antibody (both from Abcam). The precipitates were recovered by beads and analysed by qRT‐PCR.

### 2.14 Co‐immunoprecipitation

Transfected cells were lysed using RIPA, and thereafter, the collected lysates underwent immunoprecipitation using anti‐CBP, anti‐P300 or anti‐IgG (Abcam) for 1 hour, followed by the addition of Protein A‐agarose into lysates and the incubation for 12 hours. Then, complex of protein A‐agarose‐antigen‐antibody was collected through centrifugation at 12 000 *g* at 4°C for 2 minutes. After washing, precipitated proteins were tested by Western blot.

### 2.15 Western blot

Cell lysates from RIPA buffer were transferred to PVDF membranes after separation process via 10% gel electrophoresis. Samples on the membranes were sealed with 5% non‐fat dry milk for 1 hour, and the primary antibodies against CBP, P300, PCAF, HDAC7, GAPDH, MST1, MST2, p‐MST1, p‐MST2, p‐YAP1, YAP1 and corresponding anti‐IgG antibodies (all from Abcam) were used for incubate cells. At length, protein bands were detected with enhanced chemiluminescence reagent (GE Healthcare).

### 2.16 Subcellular fractionation assay

The nuclear and cytoplasmic fractions of H266 and SK‐MES‐1 cells were separated and purified as per the manual of Cytoplasmic & Nuclear RNA Purification Kit (Norgen). The isolated RNA (LINC00519, GADPH, U6) was analysed by qRT‐PCR.

### 2.17 FISH

The RNA FISH probe mix for LINC00519 was designed and synthesized by RiboBio for FISH assay in LUSC cells. Following nucleus staining using DAPI, samples were analysed utilizing laser scanning confocal microscope (ZEISS).

### 2.18 RNA immunoprecipitation

1 × 10^7^ LUSC cells (H266, SK‐MES‐1) were collected from RNA immunoprecipitation (RIP) lysis buffer and immunoprecipitated with beads conjugated to antibodies specific to Ago2 or IgG (Millipore). The precipitated complex was tested by qRT‐PCR.

### 2.19 RNA pull‐down

The protein extracts from LUSC cells were treated with biotinylated RNA (LINC00519 biotin probe) and beads for recovering, with LINC00519 no‐biotin probe as control. qRT‐PCR was operated to detect the RNA enrichment in RNA‐protein complex.

### 2.20 Dual‐luciferase reporter gene analyses

The wild type (WT) and mutant (Mut) miR‐450b‐5p or miR‐515‐5p binding sites to LINC00519 sequence or YAP1 3′‐UTR were separately cloned to pmirGLO (Promega) vectors to obtain LINC00519‐WT/Mut and YAP1‐WT/Mut vectors. The miR‐450b‐5p mimics, miR‐515‐5p mimics or NC mimics were transfected into LUSC cells with above luciferase vectors for 48 hours and finally examined using the Dual Luciferase Assay System (Promega).

### 2.21 Statistical analysis

All experimental procedures included three biological repeats. Data were statistically analysed through one‐way ANOVA and Student's *t* test by use of GraphPad Prism 6 (GraphPad), with *P* < .05 as cut‐off value. The results were presented as the mean ± SD.

## 3 RESULTS

### 3.1 Up‐regulated LINC00519 indicates unsatisfactory prognosis in LUSC

**Figure 1:**
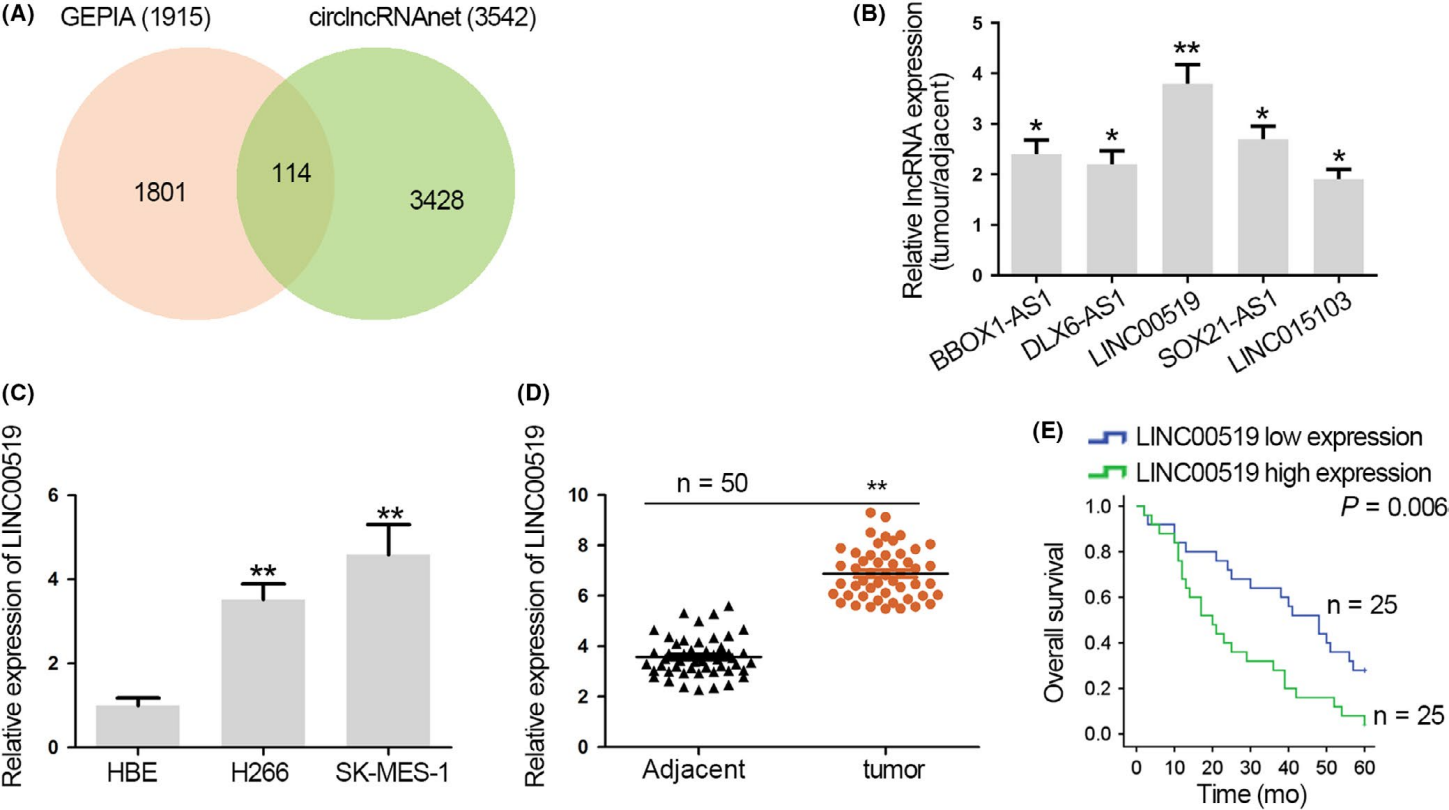
Up‐regulated LINC00519 indicates unsatisfactory prognosis in LUSC. A, The differentially expressed lncRNAs in LUSC from GEPIA and circlncRNAnet databases. B, qRT‐PCR of the expressions of the top 5 up‐regulated lncRNAs in LUSC tissues. C, qRT‐PCR of the relative LINC00519 level in H266, SK‐MES‐1 and HBE cells. D, qRT‐PCR of the relative LINC00519 level in LUSC tissues and matched adjacent tissues. E, Kaplan‐Meier method was used to analyse survival rate of LUSC patients. ^*^ *P* < .05, ^**^ *P* < .01

Based on circlncRNAnet (http://app.cgu.edu.tw/circlnc) and GEPIA (http://gepia.cancer-pku.cn/), we identified 114 lncRNAs up‐regulated in LUSC samples versus normal samples (*P* < .05, Log FC > 1) (Figure 1A). Data from qRT‐PCR showed that among 114 lncRNAs, 5 lncRNAs presented the most significant elevation in LUSC tissues (n = 3) versus correlated para‐tumour ones and LINC00519 was the top 1 up‐regulated lncRNA (Figure 1B). Therefore, we focused on LINC00519 in LUSC. We confirmed that LINC00519 expression was also higher in LUSC cells (H266, SK‐MES‐1) than that in human normal bronchial epithelial cell (HBE; Figure 1C). Additionally, we discovered that LINC00519 also showed 3‐5‐fold upregulation in lung adenocarcinoma (LUAD, another subtype of NSCLC) cells (A549 and H1299) versus normal HBE cells, which was similar to LINC00519 upregulation in LUSC cells (Figure S1A). Besides, qRT‐PCR analysis validated high LINC00519 level in 50 LUSC tissues versus the matched para‐tumour tissues (Figure 1D). Next, prognostic value of LINC00519 was assessed through Kaplan‐Meier method. As a result, LUSC patients with high LINC00519 expression showed a shorter survival time (Figure 1E). These results indicated that up‐regulated LINC00519 predicts a worse prognosis in LUSC.

### 3.2 Silenced LINC00519 restrains the progression of LUSC

To explore whether LINC00519 functioned in LUSC pathological process, loss‐of‐function assays were planned and conducted in two LUSC cell lines. Firstly, LINC00519‐specific shRNAs (sh‐LINC00519#1, sh‐LINC00519#2) were transfected into H266 and SK‐MES‐1 cells for knocking down endogenous LINC00519 expression. Expectedly, LINC00519 level was considerably reduced in LUSC cells with sh‐LINC00519#1/2 transfection (Figure S1B).

**Figure 2:**
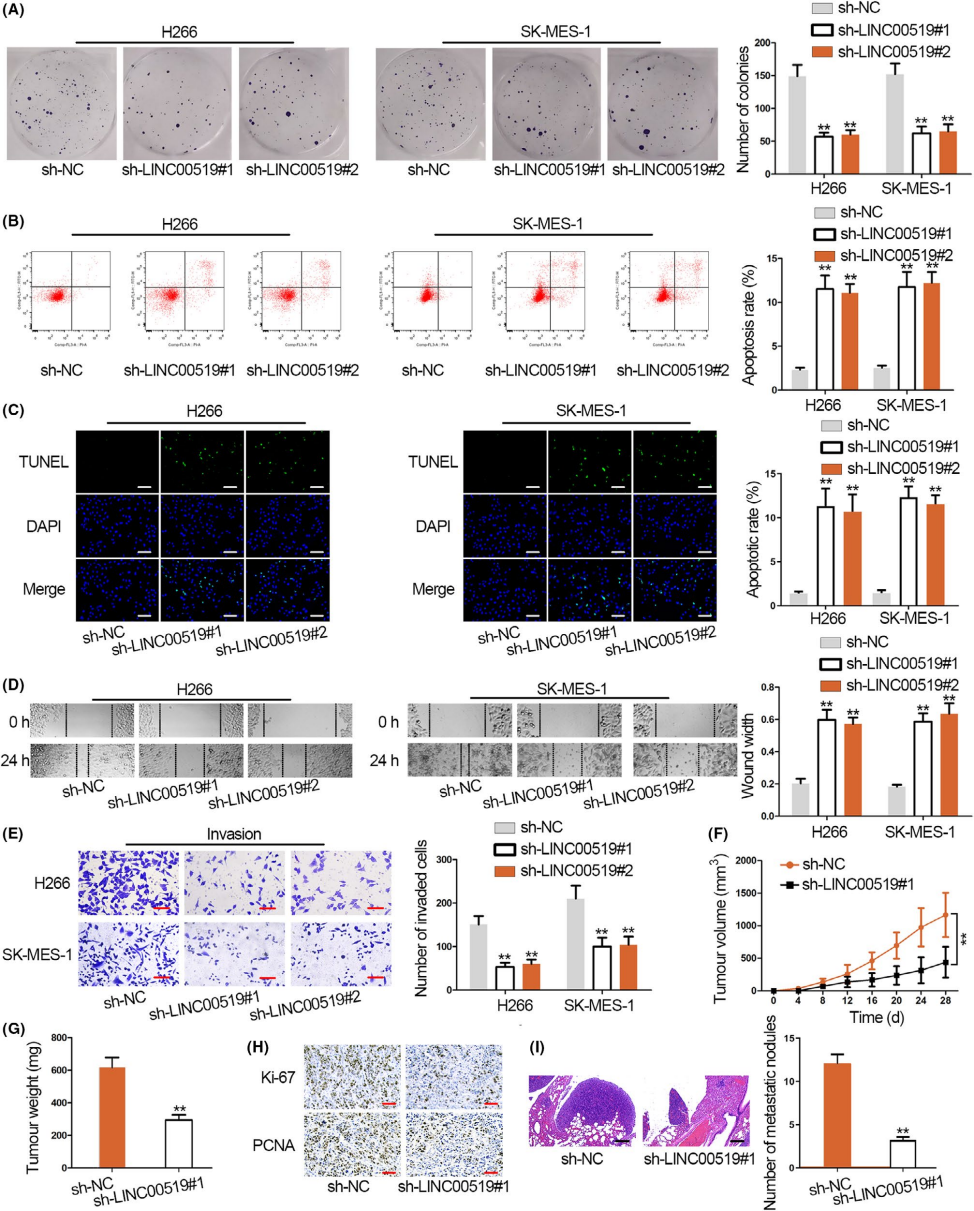
Silenced LINC00519 restrains the progression of LUSC. A, The influence of LINC00519 silence on cell proliferation was determined with colony formation assay. B,C, Cell apoptosis of sh‐LINC00519 transfected LUSC cells was assessed by flow cytometry analysis and TUNEL assay. Scale bar = 100 μm. D, The migratory capacity of LINC00519‐depleted LUSC cells was detected by conducting wound healing assay. E, Transwell assay was applied to examine the impact of LINC00519 silence on cell invasion. Scale bar = 50 μm. F,G, The effect of sh‐LINC00519 on the tumour volume and weight was observed through xenograft model. H, IHC assays showed the decreased positive immunostaining cells of Ki67 and PCNA after LINC00519 knock‐down. Scale bar = 100 μm. I, Lung metastasis model was constructed by injecting sh‐LINC00519#1 transfected LUSC cell into mice tail vein. Scale bar = 100 μm. ^**^ *P* < .01

With application of CCK‐8 kit, we observed that viable LUSC cells markedly decreased under LINC00519 from 48 hours (Figure S1C). As showed in Figure 2A, LINC00519 silence obviously reduced colonies generated by H266 and SK‐MES‐1 cells. Next, results from flow cytometry analysis and TUNEL assay showed that ratio of apoptotic LUSC cells increased by sh‐LINC00519#1/2 (Figure 2B,C). Thus, we suggested that LINC00519 could facilitate proliferation and repress apoptosis in LUSC cells.

Subsequently, we explored the effect of silenced LINC00519 on cell migration and invasion. As shown in Figure 2D, the wound width in H266 and SK‐MES‐1 cells was wider in sh‐LINC00519#1/2 groups than in sh‐NC group at 24 hours, indicating that the migratory ability was alleviated in LUSC cells transfected with sh‐LINC00519#1/2. Besides, LUAC cells invading to the lower transwell chamber was less in sh‐LINC00519#1/2 groups than in sh‐NC group (Figure 2E), indicating that LINC00519 knock‐down effectively weakened the invasive capacity of LUSC cells.

Further, to monitor the role of LINC00519 in tumour growth, we conducted in vivo experiments. LINC00519‐depleted SK‐MES‐1 cells were injected into the nude mice. Then, it was discovered that LINC00519 depletion efficiently reduced tumour volume and weight (Figure 2F,G and Figure S1D). IHC assay demonstrated that the expressions of Ki‐67 and PCNA (cell proliferation markers) were significantly down‐regulated in LINC00519‐silenced tumours (Figure 2H). As displayed in lung metastasis model, LINC00519 knock‐down remarkably decreased the number of metastatic nodules which formed in mouse lungs (Figure 2I). Taken together, silenced LINC00519 restrains LUSC progression.

### 3.3 CBP/P300 complex‐mediated H3K27ac activated LINC00519 expression

**Figure 3:**
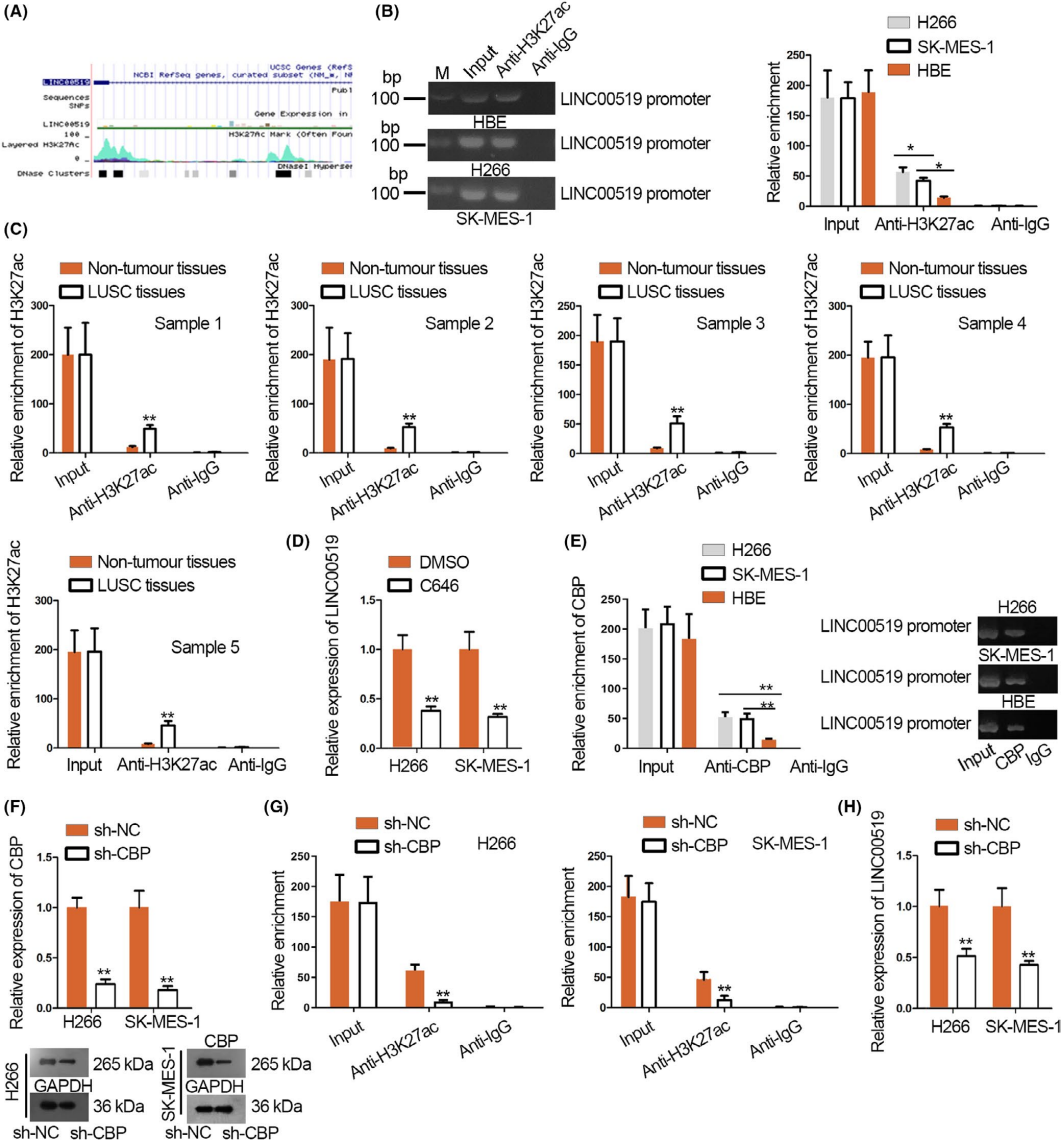
CBP/P300‐mediated H3K27ac activated LINC00519 expression. A, The enrichment of H3K27ac at LINC00519 promoter was demonstrated in UCSC. B, ChIP assay was utilized to assess H3K27ac enrichment at LINC00519 promoter in H266, SK‐MES‐1 and HBE cells. C, ChIP assay was utilized to assess H3K27ac enrichment at LINC00519 promoter in five LUSC tissues and five matched normal tissues. D, qRT‐PCR analysis was conducted to measure LINC00519 expression in LUSC cells introduced with DMSO control or C646. E, The enrichment of LINC00519 promoter by CBP in LUSC cells and HBE cell was evaluated via ChIP assay. F, The efficiency of sh‐CBP was estimated with qRT‐PCR and Western blotting. G, The effect of CBP knock‐down on H3K27ac enrichment was assessed. H, LINC00519 expression in sh‐CBP transfected cells was presented by qRT‐PCR. ^*^ *P* < .05, ^**^ *P* < .01

Increasing studies have confirmed that abnormal expression of lncRNAs resulted from transcriptional activation, which can be influenced by histone acetylation.^16^ To investigate how LINC00519 was up‐regulated in LUSC, we searched UCSC (http://genome.ucsc.edu/). It was found that the LINC00519 promoter region was dramatically enriched with H3K27ac (Figure 3A). To confirm this, we conducted ChIP assay with the use of anti‐H3K27ac antibody. Data implied that the enrichment of H3K27ac in LINC00519 promoter region was higher in H266 and SK‐MES‐1 cells than in HBE cells (Figure 3B). We also conducted ChIP assay based on 5 LINC00519 up‐regulated LUSC tissues and 5 paired non‐tumour tissues. As expected, high H3K27ac enrichment in LINC00519 promoter region was observed in LUSC tissues (Figure 3C). To test whether H3K27ac was specific for LINC00519 transcription activation and expression upregulation, we treated LUSC cells with C646, the histone acetyltransferase inhibitor. Consequently, LINC00519 expression declined in H266 and SK‐MES‐1 cells treated with C646 (Figure 3D). Also, luciferase activity of LINC00519 promoter was repressed markedly by C646 in LUSC cells (Figure S2A), indicating that H3K27ac was related to LINC00519 expression and transcription in LUSC cells.

Subsequently, we investigated how H3K27ac in LINC00519 promoter was regulated. CBP was reported as an essential factor for chromatin acetylation,^17^ so we speculated that CBP might participate in the improved acetylation. To verify this speculation, we tested whether CBP bound to LINC00519 promoter by ChIP. The results depicted that a considerable enrichment of LINC00519 promoter in ChIP products of CBP antibody, and the enrichment level was markedly higher in LUSC cells than HBE cells (Figure 3E). To estimate the effect of CBP on H3K27ac, CBP mRNA and protein levels were knocked down by sh‐CBP (Figure 3F and Figure S5A). As expected, CBP deficiency caused an observable decrease of H3K27ac enrichment in LINC00519 promoter in H266 and SK‐MES‐1 cells (Figure 3G). CBP silence significantly reduced LINC00519 expression in LUSC cells (Figure 3H).

Moreover, CBP is known to form a complex with P300, another histone acetyltransferase, to regulate H3K27ac,^18^, ^19^ so we tested the interaction between them in LUSC cells. Co‐immunoprecipitation (CoIP) data confirmed the abundant CPB protein in P300 precipitates and the enriched P300 protein in CBP precipitates (Figure S2B). We also knocked down P300 in 2 LUSC cells and confirmed the decreased endogenous P300 mRNA and protein after transfection of sh‐P300 (Figure S2C and Figure S5D). Consistently, P300 knock‐down reduced H3K27ac in LINC00519 promoter according to ChIP data (Figure S2D), and LINC00519 level declined upon P300 silence in LUSC cells as well (Figure S2E). Besides, to preliminarily determine whether CBP/P300 was the crucial factor for H3K27ac in LINC00519 promoter, we tested other acetyltransferases reported to regulate H3K27ac, including HDAC7^20^ and PCAF.^21^ We first validated the knock‐down of HDAC7 and PCAF in LUSC cells after transfecting specific shRNAs (Figure S2F and Figure S5E). However, we discovered that knocking down either HDAC7 or PCAF failed to alter the luciferase activity of LINC00519 promoter activity and expression in LUSC cells (Figure S2G,H), indicating that CBP/P300 might be vital for promoter H3K27ac and upregulation of LINC00519 in LUSC cells.

Additionally, we tested whether CBP/P300 regulated LINC00519 transcription and expression via H3K27ac in LUAD cells as well. We discovered that similar to LUSC cells, H3K27ac enrichment in LINC00519 promoter was higher in LUAD cell lines (A549 and H1299) versus normal HBE cells (Figure S3A). Then, we knocked down P300 and CBP in LUAD cell lines by shRNAs and confirmed the decrease of P300 and CBP proteins by sh‐P300 and sh‐CBP (Figure S3B and Figure S5F). As expected, knocking down either P300 or CBP reduced LINC00519 level in LUAD cells (Figure S3C). Accordingly, less LINC00519 promoter was enriched in the ChIP products of H3K27ac (Figure S3D), indicating that CBP/P300 regulated H3K27ac in LINC00519 promoter in LUAD cells. Moreover, either P300 or CBP knock‐down repressed activity of LINC00519 promoter reporter IN LUAD cells (Figure S3E). In brief, all results above suggested that CBP‐mediated H3K27ac could activate the expression of LINC00519.

### 3.4 LINC00519 regulates YAP1 in LUSC

**Figure 4:**
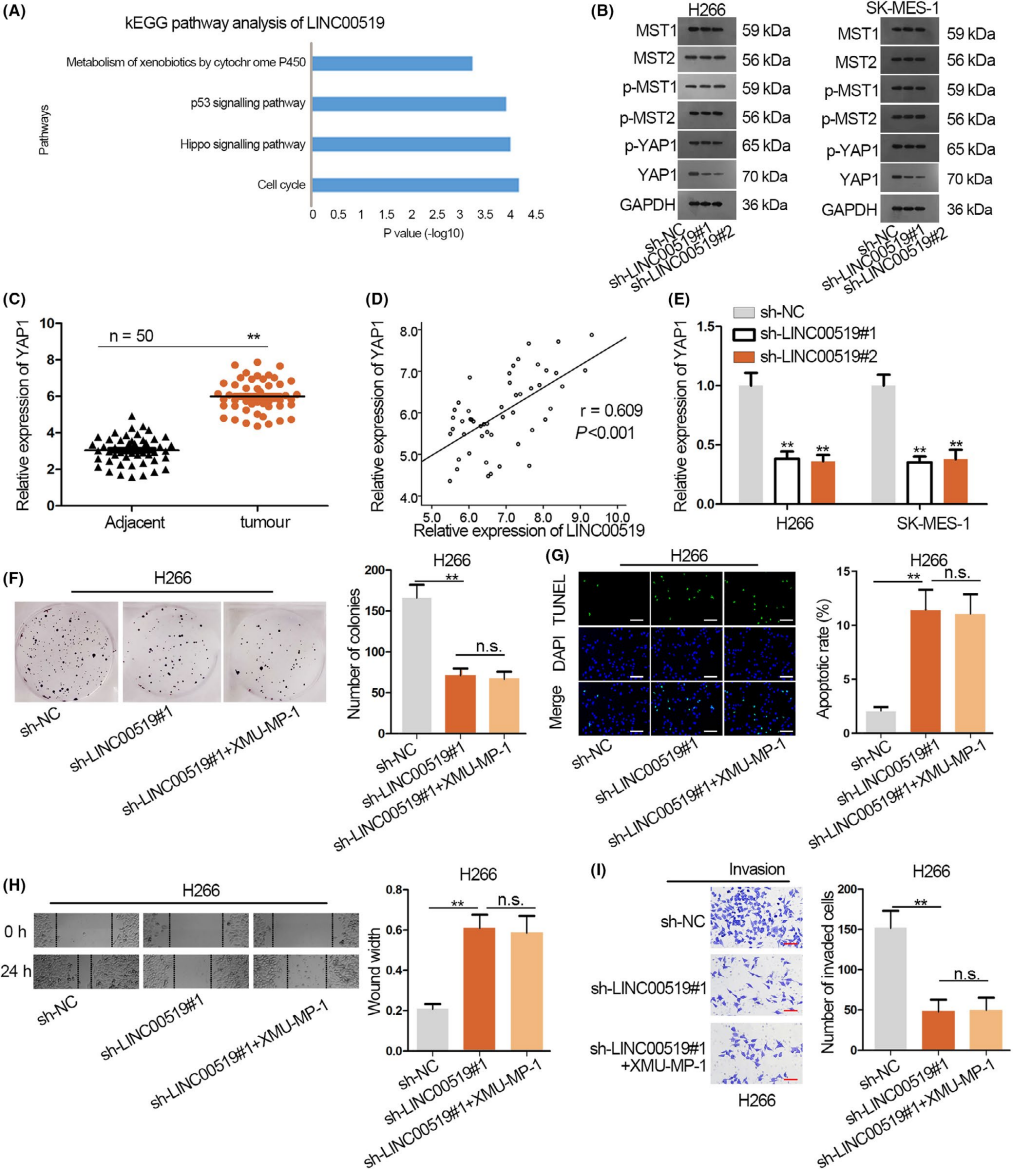
LINC00519 regulates YAP1 in LUSC. A, The involvement of LINC00519 in Hippo pathway was showed in circlncRNAnet through KEGG pathway. B, The effect of LINC00519 down‐regulation on the protein levels of MST1/2, p‐MST1/2, p‐YAP1 and YAP1 was estimated by Western blot assay. C, qRT‐PCR of YAP1 expression in LUSC tissues and paired non‐tumour tissues. D, The relevance between LINC00519 and YAP1 was analysed by Spearman's correlation analysis. E, YAP1 expression in LUSC cells transfected with sh‐LINC00519. F, The effect of XMU‐MP‐1 on LUSC cell proliferation after transfecting sh‐LINC00519#1. G, The pictures and quantification of TUNEL‐labelled LUSC cells which was transfected with sh‐LINC00519#1 and XMU‐MP‐1. Scale bar = 100 μm. H, sh‐LINC00519#1 and XMU‐MP‐1 was introduced to LUSC cells to test the migration in wound healing experiment. I, Efficiency of silenced LINC00519 and XMU‐MP‐1 on cell invasion was analysed in transwell system. Scale bar = 50 μm. ^**^ *P* < .01

Next, circlncRNAnet showed that LINC00519 was involved in Hippo pathway through Kyoto Encyclopedia of Genes and Genomes (KEGG) pathway (Figure 4A). To check this prediction, the key factors in Hippo pathway, including MST1/2, p‐MST1/2, p‐YAP1 and YAP1, were detected in LINC00519 silenced LUSC cells. Consequently, LINC00519 knock‐down decreased only YAP1 protein level but failed to alter the levels of MST1/2, p‐MST1/2 and p‐YAP1 (Figure 4B and Figure S5B), indicating that LINC00519 positively regulated YAP1. YAP1 is a pivotal factor in Hippo pathway, and when Hippo signalling is turned off, YAP1 is activated to elicit carcinogenic functions in cancers including lung cancer.^22^, ^23^ High YAP1 expression in LUSC tissues was confirmed (Figure 4C). Notably, the LINC00519 and YAP1 expressions presented positive correlation in LUSC samples as analysed by Spearman's correlation method (Figure 4D). qRT‐PCR analysis delineated the diminished YAP1 expression with the transfection of sh‐LINC00519 (Figure 4E). Additionally, XMU‐MP‐1 (a Hippo pathway inhibitor which inhibits MST1/2) was employed for the rescue assays. As presented in Figure 4F, the treatment of XMU‐MP‐1 failed to alter the inhibitive effect of silenced LINC00519 on cell proliferation. Besides, the facilitated apoptosis caused by LINC00519 knock‐down was not changed under XMU‐MP‐1 treatment (Figure 4G). Moreover, XMU‐MP‐1 did not recover the migration and invasion that were suppressed in LINC00519 down‐regulated cells (Figure 4H,I). Above data suggested that LINC00519 circumvents the upstream MST1/2 in Hippo pathway to directly regulate YAP1 in LUSC.

### 3.5 LINC00519 up‐regulates YAP1 by sponging miR‐450b‐5p and miR‐515‐5p in LUSC

**Figure 5:**
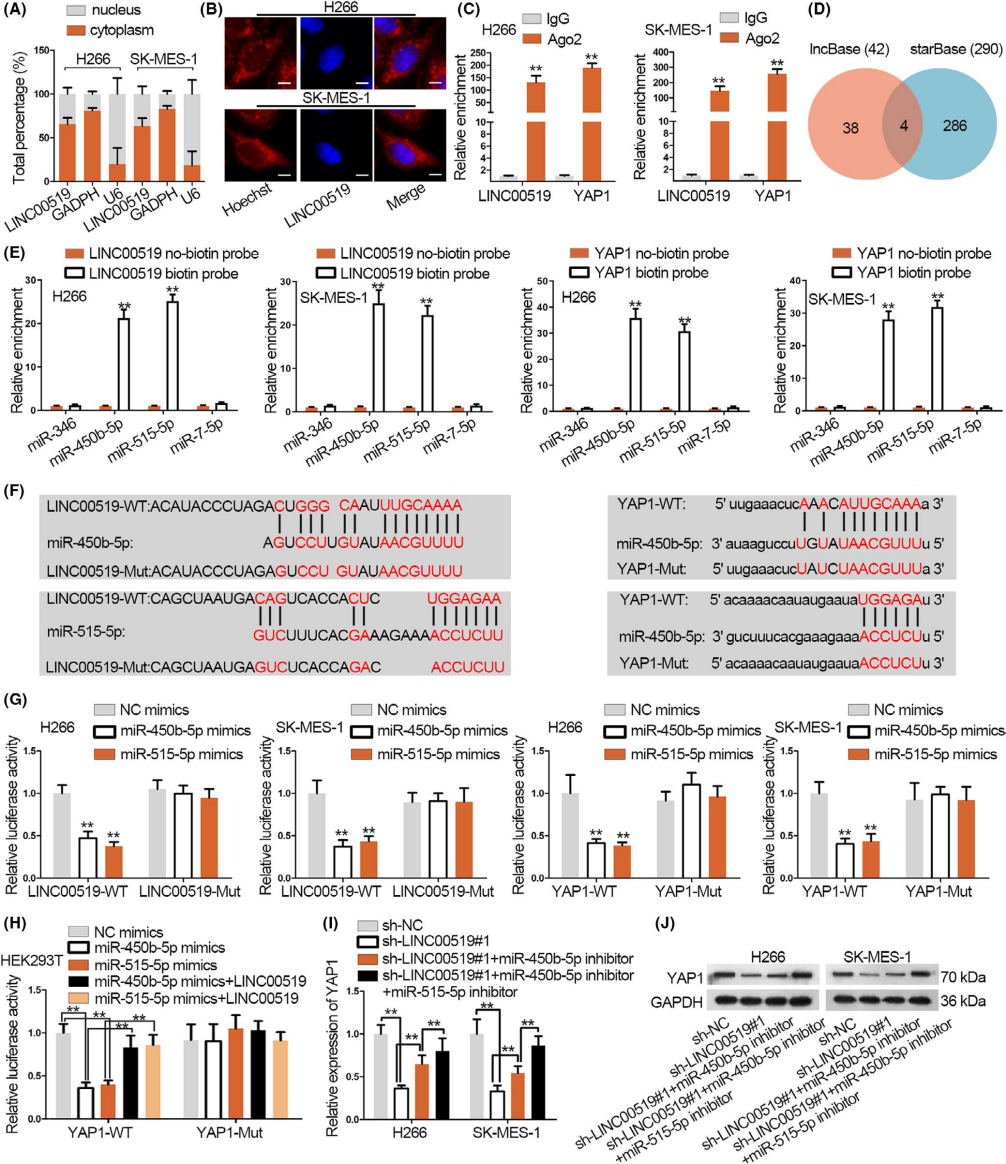
LINC00519 up‐regulates YAP1 by sponging miR‐450b‐5p and miR‐515‐5p in LUSC. A,B, The location of LINC00519 was determined in LUSC cells via subcellular fractionation and FISH assays. Scale bar = 10 μm. C, The enrichments of LINC00519 and YAP1 in the beads conjugated with Ago2 antibody were determined by performing RIP assays. D, The potential miRNAs for LINC00519 and YAP1. E, The interaction between potential miRNAs and LINC00519/YAP1 was validated with RNA pull‐down assay. F, The miR‐450b‐5p/miR‐515‐5p binding sites for LINC00519 and YAP1 were predicted and mutated sites were designed. G, Luciferase reporter LINC00519/YAP1‐WT and LINC00519/YAP1‐Mut were constructed to confirm the binding between LINC00519/YAP1 and miR‐450b‐5p/miR‐515‐5p. H, The luciferase activity of YAP1‐WT/YAP1‐WT/Mut in HEK293T cell was validated. I,J, YAP1 mRNA and protein levels were detected in LUSC cells which were transfected the indicated plasmids. ^**^ *P* < .01

Thereafter, we interrogated how LINC00519 regulated YAP1. We first carried out the subcellular fractionation assay, finding that LINC00519 was mainly distributed in the cytoplasm of H266 and SK‐MES‐1 cells (Figure 5A). FISH assay also verified that LINC00519 was a cytoplasmic RNA (Figure 5B). Through RIP assay, both LINC00519 and YAP1 mRNA were enriched in the RIP of Ago2 rather than IgG (Figure 5C). Taken together, we hypothesized that LINC00519 might function as a ceRNA to up‐regulate YAP1 by sponging miRNAs. Thus, we planned to search the underlying miRNAs for LINC00519 and YAP1. 4 miRNAs (miR‐346, miR‐450b‐5p, miR‐515‐5p and miR‐7‐5p) were screened out from LncBase (http://carolina.imis.athenainnovation.gr/diana-tools/web/index.php?r=lncbasev2/index) and starBase (http://starbase.sysu.edu.cn/index.php) (Figure 5D). According to RNA pull‐down assay, we found that miR‐450b‐5p and miR‐515‐5p were pulled down by LINC00519 biotin probe and YAP1 biotin probe (Figure 5E). Hence, we speculated miR‐450b‐5p and miR‐515‐5p as target miRNAs for LINC00519 to regulate YAP1 in LUAC. The binding sites for 2 miRNAs in between LINC00519 and YAP1 (LINC00519‐WT/YAP1‐WT) as well as the mutated sites (LINC00519‐Mut/YAP1‐Mut) were presented in Figure 5F. Next, we transfected miR‐450b‐5p mimics and miR‐515‐5p mimics, respectively, into LUSC cells and verified the overexpression of miR‐450b‐5p and miR‐515‐5p (Figure S4A). We discovered that miR‐450b‐5p mimics and miR‐515‐5p mimics decreased the luciferase activity of LINC00519‐WT/YAP1‐WT rather than LINC00519‐Mut/YAP1‐Mut (Figure 5G). Besides, LINC00519 was validated to be overexpressed in LUSC cells by pcDNA3.1/LINC00519 (Figure S4B). For the easy transfection and small derivation of experimental data, HEK293T cells were applied to conduct the experiment. Data revealed that LINC00519 overexpression restored luciferase activity of YAP1‐WT that was repressed by miR‐450b‐5p mimics/miR‐515‐5p mimics whereas YAP1‐Mut was hardly changed (Figure 5H). Later, we validated that miR‐450b‐5p inhibitors and miR‐515‐5p inhibitors significantly reduced miR‐450b‐5p and miR‐515‐5p expressions, respectively, in LUSC cells (Figure S4C). Finally, mRNA and protein levels of YAP1 down‐regulated by LINC00519 silence were partially reserved by miR‐450b‐5p inhibitor, and the co‐transfection of miR‐450b‐5p and miR‐515‐5p inhibitors nearly fully restored the effect of LINC00519 silence (Figure 5I‐J and Figure S5C). In conclusion, LINC00519 up‐regulates YAP1 through sponging miR‐450b‐5p and miR‐515‐5p in LUSC.

### 3.6 MiR‐450b‐5p and miR‐515‐5p inhibit LUSC cell growth and metastasis

**Figure 6:**
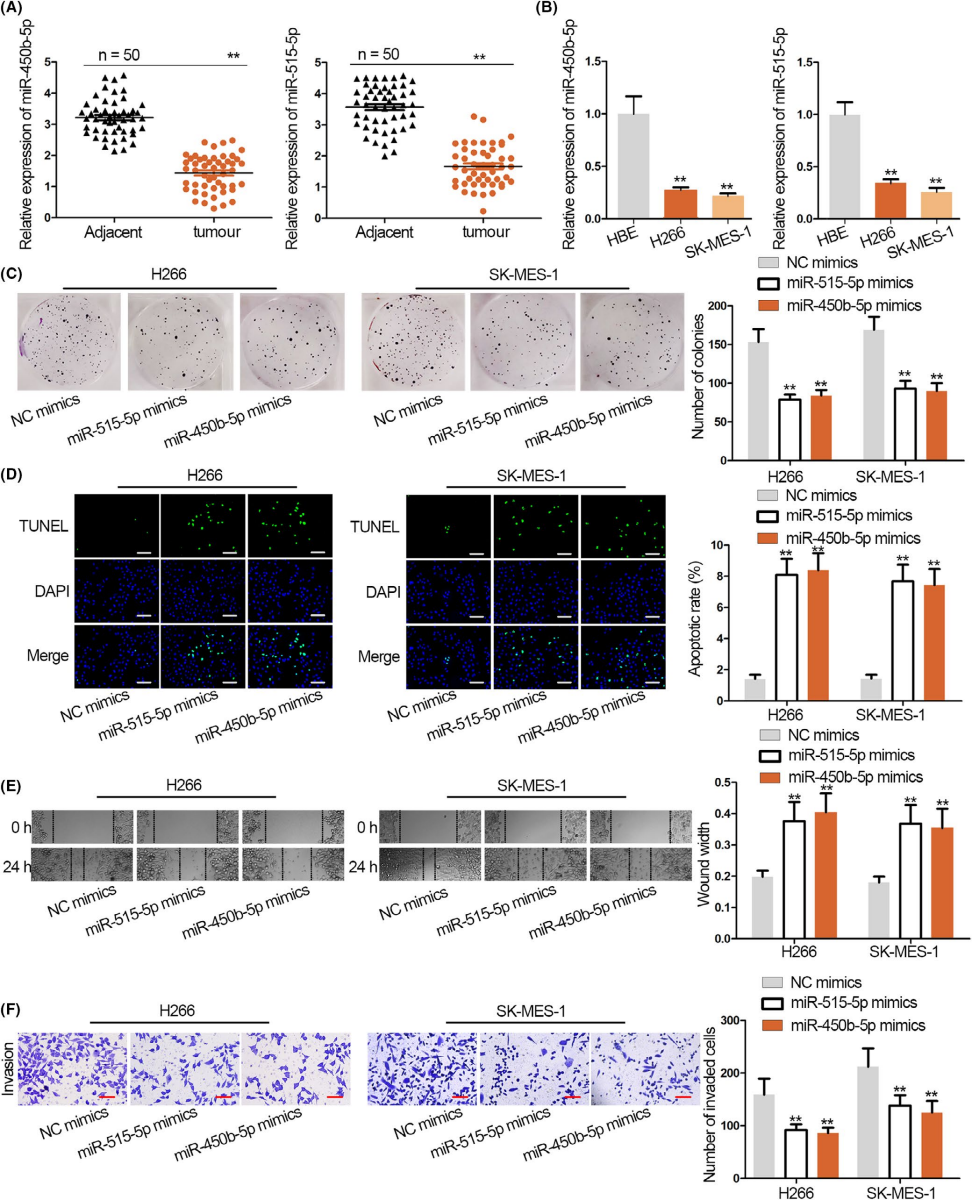
MiR‐450b‐5p and miR‐515‐5p inhibit LUSC cell growth. A, qRT‐PCR of miR‐450b‐5p and miR‐515‐5p expressions in LUSC tissues and normal adjacent tissues. B, qRT‐PCR of miR‐450b‐5p and miR‐515‐5p expressions in LUSC cells and HBE cells. C, The proliferation in miR‐450b‐5p mimics and miR‐515‐5p mimics transfected LUSC cells was assessed by colony formation assay. D, TUNEL assay demonstrated the effect of overexpressed miR‐450b‐5p and miR‐515‐5p on cell apoptosis. Scale bar = 100 μm. E, Impacts of miR‐450b‐5p and miR‐515‐5p overexpression on the migratory ability of LUSC cells was analysed by wound healing assay. F, The invasion in miR‐450b‐5p and miR‐515‐5p overexpressed cells was evaluated by transwell assay. Scale bar = 50 μm. ^**^ *P* < .01

Previous studies have uncovered the anti‐carcinogenic role of miR‐450b‐5p and miR‐515‐5p in some cancers, including in lung cancer.^24^, ^25^, ^26^, ^27^ Nonetheless, their specific functions in LUSC cells are still unclear, so we tested the effects of miR‐450b‐5p and miR‐515‐5p in LUSC cells. We discovered that miR‐450b‐5p and miR‐515‐5p were under‐expressed in LUSC tissues and cell lines (Figure 6A,B). Then, colony formation, TUNEL, wound healing and transwell assays were conducted to identify the biological function of miR‐450b‐5p and miR‐515‐5p on LUSC cell growth and metastasis. As shown in Figure 6C, miR‐450b‐5p mimics and miR‐515‐5p mimics restrained cell proliferation. The apoptosis of H266 and SK‐MES‐1 cells was facilitated by miR‐450b‐5p mimics and miR‐515‐5p mimics (Figure 6D). Based on the result of wound healing assay, cell migration was suppressed by the overexpression of miR‐450b‐5p and miR‐515‐5p (Figure 6E). At last, transwell assay revealed the repressive effect of miR‐450b‐5p and miR‐515‐5p overexpression on the invasive capability of H266 and SK‐MES‐1 cells (Figure 6F). Namely, miR‐450b‐5p and miR‐515‐5p inhibit LUSC cell growth and metastasis in vitro.

### 3.7 LINC00519 boosts LUSC cell growth through up‐regulating YAP1

**Figure 7:**
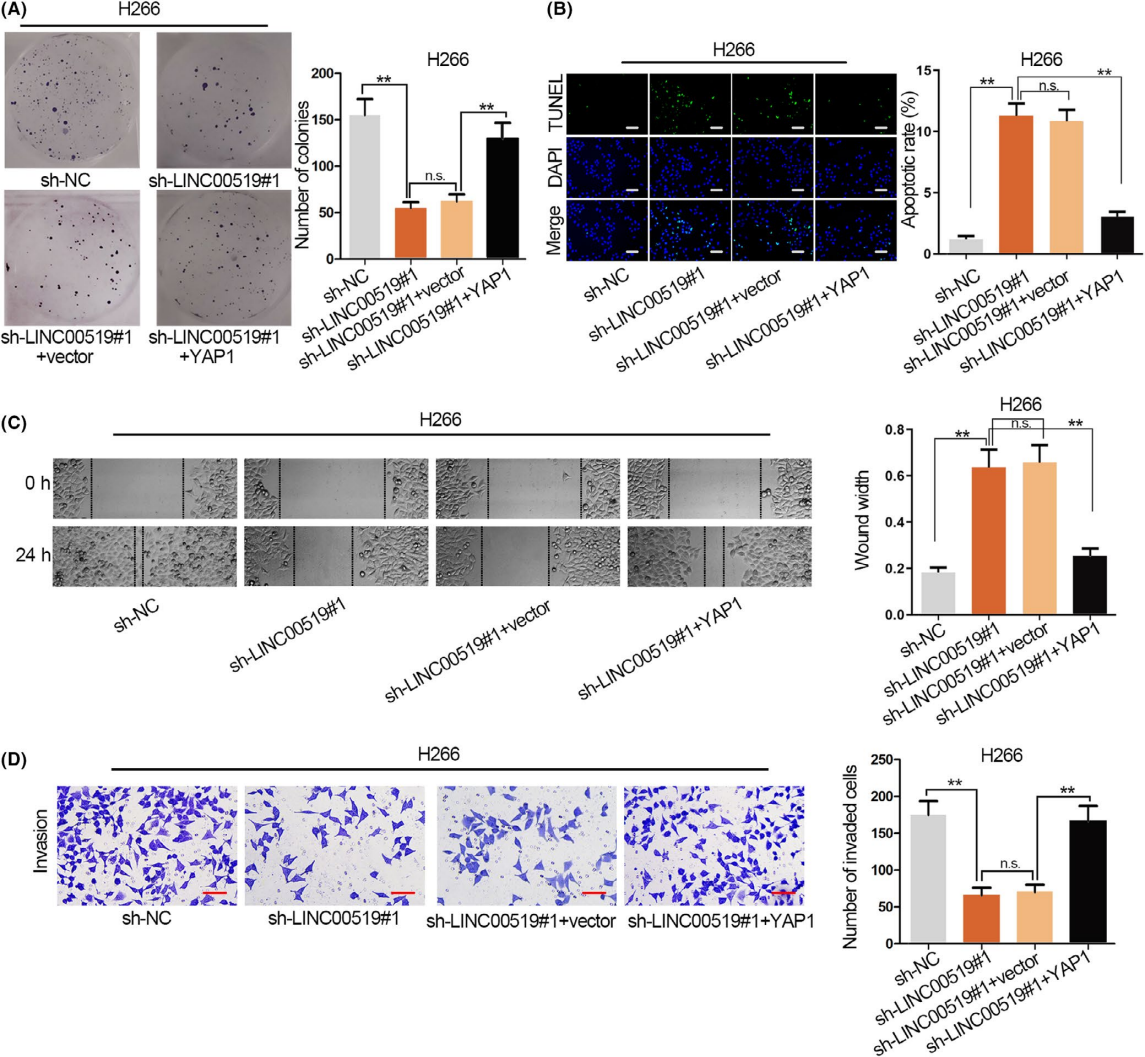
LINC00519 aggravates LUSC progression through up‐regulating YAP1. A, Proliferation ability was detected in H266 cells transfected with sh‐LINC00519#1 and YAP1. B, The apoptosis in H266 cells which was co‐transfected with sh‐LINC00519#1 and YAP1. Scale bar = 100 μm. C, Cell migration was examined after sh‐LINC00519#1 and YAP1 were transfected to H266 cells. D, The effect of sh‐LINC00519#1 and sh‐LINC00519#1 + YAP1 cell invasion was determined by transwell assay. Scale bar = 50 μm. ^**^ *P* < .01. n.s. indicated no significance

To further analyse whether LINC00519 exerted oncogenic role in LUSC via regulating YAP1, we designed and carried out a series of restoration experiments in H266 cells. YAP1 expression was up‐regulated in H266 cells (Figure S4D). The result of colony formation assay depicted that cell proliferation weakened by LINC00519 depletion was counteracted by overexpressed YAP1 (Figure 7A). The elevated cell apoptosis resulted from silenced LINC00519 was reversed by YAP1 overexpression (Figure 7B). Meanwhile, sh‐LINC00519#1‐mediated suppression on cell migration was offset by the up‐regulation of YAP1 (Figure 7C). Finally, up‐regulated YAP1 recovered the repressive effect of silenced LINC00519 on cell invasion (Figure 7D). Therefore, we confirmed that LINC00519 aggravates LUSC cell proliferation, migration and invasion as well as suppresses cell apoptosis through up‐regulating YAP1.

## 4 DISCUSSION

Previous studies have showed the regulatory role of lncRNAs in human diseases, particularly in cancers.^28^, ^29^ It is reported that lncRNAs regulated tumour progression via diverse mechanisms. For example, lncRNA MALAT1 modulates gastric cancer cell autophagy via sequestrating miR‐23b‐3p.^30^ LncRNA HOXB‐AS3A encodes peptide to restrain cell growth in colon cancer growth.^31^ LncRNA IGFBP4‐1 is overexpressed and promotes lung cancer cell proliferation by reprogramming energy metabolism.^32^ This study first identified a novel lncRNA up‐regulated in LUSC based on data analysis in GEPIA and circlncRNAnet. We confirmed the elevation of LINC00519 level in LUSC tissues and cells, indicating the link between LINC00519 and LUSC. Besides, we suggested the prognostic value of LINC00519 by revealing that high LINC00519 level indicated the unfavourable prognosis in LUSC patients. In vitro functional experiments depicted that silenced LINC00519 prohibited proliferation, migration, invasion and stimulated apoptosis in LUSC cells. In vivo data demonstrated that LINC00519 knock‐down inhibited tumour growth and lung metastasis. These results indicated the oncogenic property of LINC00519 in LUSC.

H3K27ac was firstly found in yeast.^33^ Long pliable N‐terminal tails in histone proteins could be subject to numerous covalent modifications, containing acetylation.^34^ In addition, acetylation weakens DNA‐histone interaction by neutralizing positive charge of Lys and thereby activated transcription.^35^ With the great improvement in DNA sequencing technology, it was possible to analyse the distribution patterns of histone acetylation through the whole genome.^36^ Former researches have confirmed the association between histone H3K27ac dysregulation and tumour progression.^37^ For example, lncRNA GHET1 is verified to be up‐regulated and activated by H3K27ac at promoter region.^38^ Herein, genome bioinformatics analysis in UCSC showed that H3K27ac was abundant at LINC00519 promoter region, suggesting that LINC00519 might be transcriptionally activated by histone acetylation in LUSC. As expected, we discovered that H3K27ac level in LINC00519 promoter was higher in LUSC cells and tissues. In detail, we uncovered that CBP/P300, the histone acetyltransferase complex renowned for H3K27ac,^18^, ^19^ induced LINC00519 transcription and expression by increasing H3K27ac in the promoter region. However, other histone acetyltransferases such as PCAF and HDAC7 failed to affect LINC00519 promoter transcription. These data suggested that CBP/P300 complex was the vital factor that induces H3K27ac and transcription of LINC00519 promoter, leading to LINC00519 upregulation in LUSC. Additionally, we also confirmed that CBP/P300 had same impact on lung adenocarcinoma (LUAD) cells, indicating that LINC00519 upregulation was universally attributed to CBP/P300 in NSCLC.

YAP1 is an essential downstream gene of Hippo pathway and has been reported as a carcinogen in cancers. For instance, YAP1 promotes the autophagy in sarcoma.^39^ YAP1 is reported to be overexpressed in thyroid papillary carcinoma.^40^ In our presented study, although KEGG pathway analysis showed the link between LINC00519 and hippo pathway, we found that LINC00519 cannot affect MTS1/2 but up‐regulated YAP1. Moreover, rescue assays using MTS1/2 inhibitor indicated that LINC00519 directly up‐regulated YAP1 without influencing upstream factors in hippo pathway.

Subsequently, we found that LINC00519 is mainly localized in the cytoplasm of LUSC cells, suggesting the ceRNA function of LINC00519 in LUSC. Next, LINC00519 and YAP1 were verified to interact with RISC, which confirmed ceRNA hypothesis. It is commonly acknowledged that lncRNAs exert regulatory functions through acting as ceRNAs which modulate mRNA expression by sponging miRNAs. For instance, lncRNA UICLM acts as a ceRNA to regulate ZEB2 expression and trigger tumour growth and liver metastasis by sponging miRNA‐215 in colorectal cancer.^41^ LncRNA CRNDE acts as a ceRNA to up‐regulate IRS1 expression and facilitate cell proliferation and metastasis through sponging miR‐384 in pancreatic cancer.^42^ LncRNA RP11‐436H11.5 acts as a ceRNA to up‐regulate BCL‐W expression and promote the proliferation and invasion via sponging miR‐335‐5p in renal cell carcinoma.^30^ Herein, we first validated that miR‐450b‐5p and miR‐515‐5p combined with LINC00519 and YAP1 in LUSC. Besides, miR‐450b‐5p and miR‐515‐5p expressions were evidently down‐regulated in LUSC tissues and cells, and played suppressive role in LUSC cells by repressing proliferation, migration, invasion and stimulating role in cell apoptosis. Restoration experiments revealed that YAP1 overexpression could rescue LINC00519 silencing‐mediated inhibition on LUSC cell growth, suggesting YAP1 was a target for LINC00519 to regulate LUSC progression.

**Figure 8:**
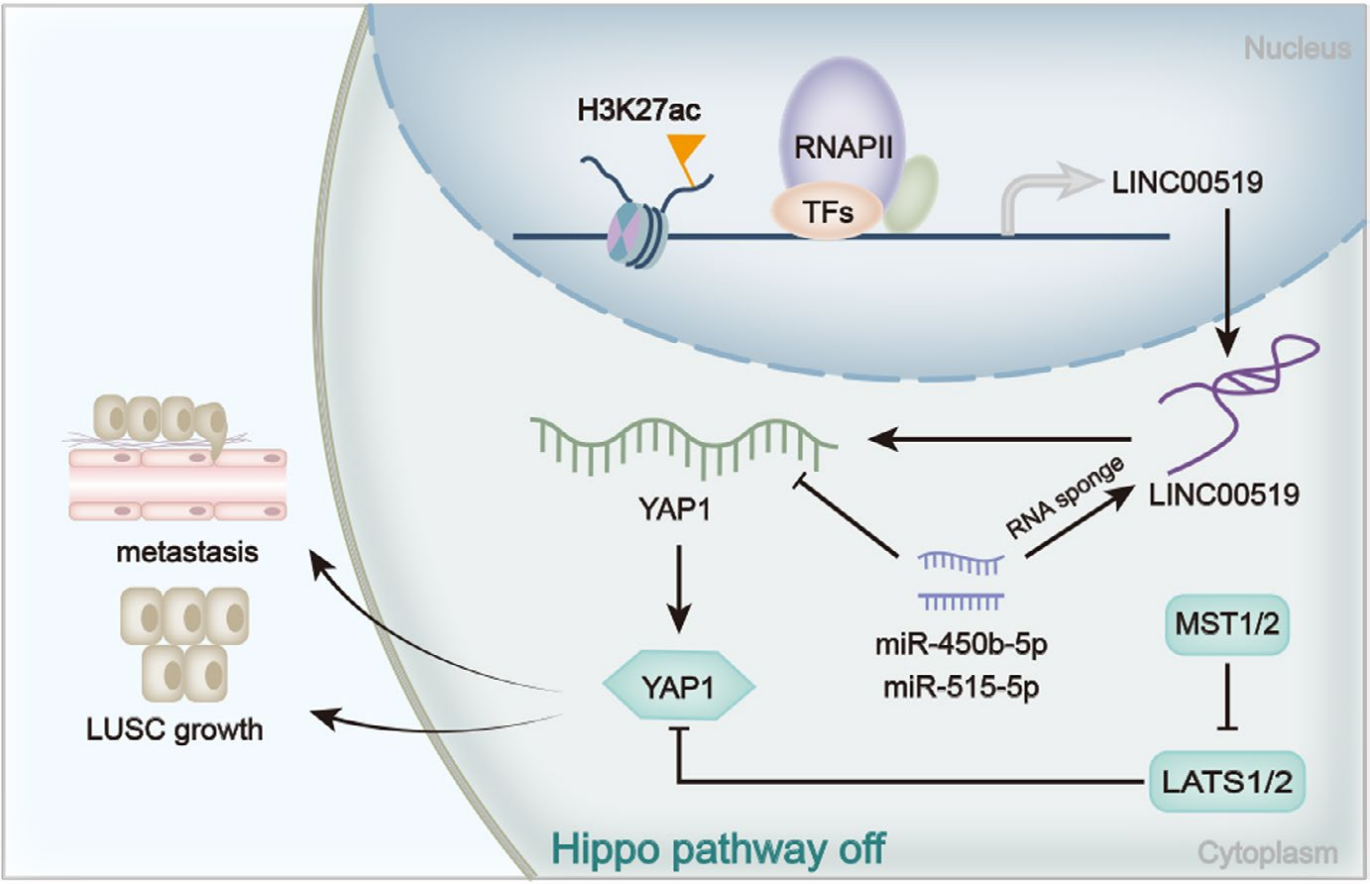
H3K27ac‐LINC00519‐miR‐450b‐5p/miR‐515‐5p‐YAP1 axis in LUSC was generated and illustrate by mechanism diagram

In conclusion, the oncogenic role of LINC00519 was first revealed in this study, and H3K27ac‐activated LINC00519 promotes LUSC progression by targeting miR‐450b‐5p/miR‐515‐5p and regulating YAP1 (Figure 8). This research might provide a meaningful revelation for exploring novel LUSC therapeutic method. However, other factors that might influence the pathway we demonstrated in this study is not investigated here, and we will dedicate ourselves to further explore the interaction of this pathway with other factors in LUSC in the near future.

## CONFLICTS OF INTEREST

Authors state no conflicts of interest in this study.

## AUTHORS’ CONTRIBUTIONS

Peng Ye, Xiayi Lv and Jun Cheng: conceptualization, investigation and project administration. Rusidanmu Aizemaiti: data curation. Peng Ye and Xiayi Lv: formal analysis, supervision and validation. Pinghui Xia and Di Meng: writing‐original draft, writing‐review and editing. All authors approved final manuscript.

## ETHICAL APPROVAL

Experiments involved in patients performed with the signed informed consents from all participants and the approval from the Ethical Review Board of the First Affiliated Hospital of Zhejiang University. This animal study was approved and performed strictly in accordance with the Institutional Guidelines on the Use of Live Animals in the First Affiliated Hospital of Zhejiang University.

## Supporting information

Fig S1‐S5

## Data Availability

Research data are not shared.
