# Supplementary material for: H3K27ac‐activated LINC00519 promotes lung squamous cell carcinoma progression by targeting miR‐450b‐5p/miR‐515‐5p/YAP1 axis
Source: Cell Prolif. 2020 Apr 16;53(5):e12797. doi: 10.1111/cpr.12797 (PMC7260072; doi:10.1111/cpr.12797)

**Fig. S1 Supplementary data for figure 1 and 2.** (A) qRT-PCR of LINC00519 level in LUAD cells (H1299 and A549) versus normal HBE cells. (B) LINC00519 was silenced in H266 and SK-MES-1 cells by transfecting sh-LINC00519. (C) Proliferation of H266 and SK-MES-1 cells under LINC00519 knockdown was tested by CCK-8. (D) Pictures of xenografts in mice subcutaneously injected H266 cells with LINC00519 knockdown versus control. ^**^P < 0.01.


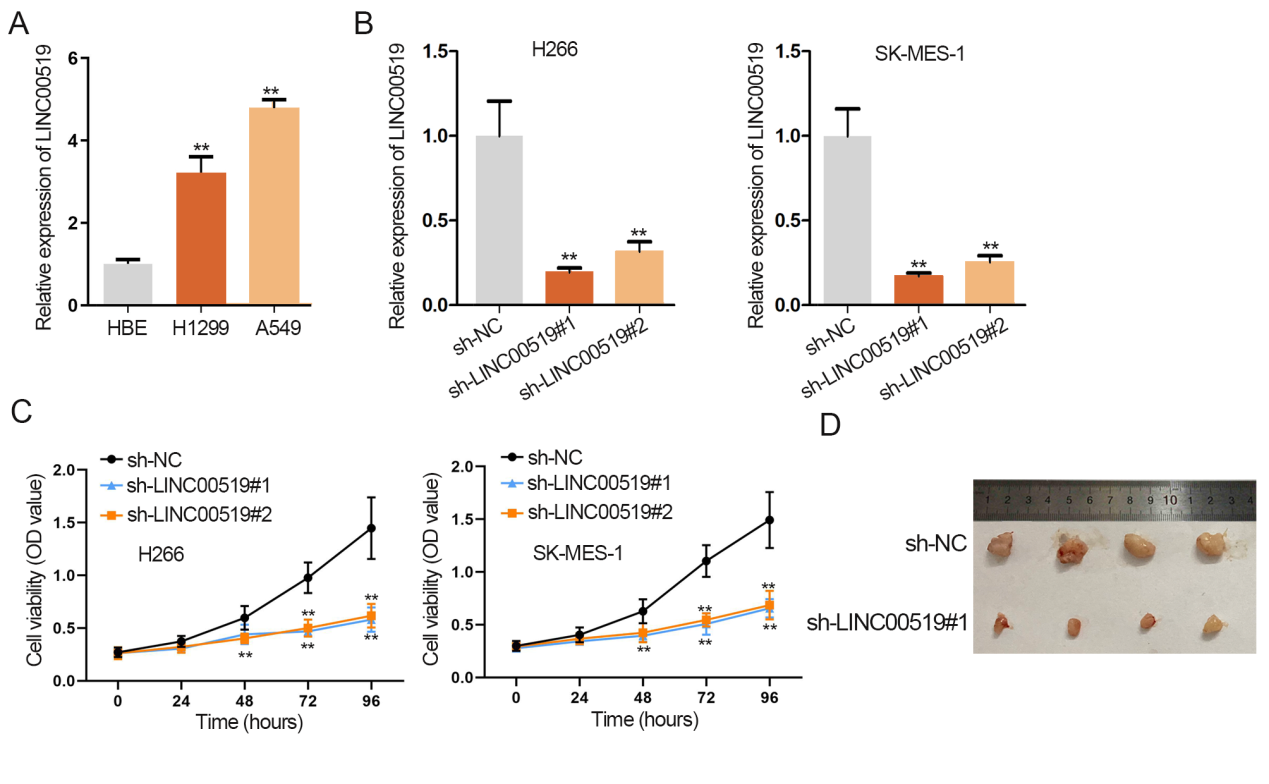


**Fig. S2 CBP/P300 was vital for H3K27ac in LINC00519 promoter.** (A) Luciferase activity of LINC00519 promoter reporter in LUSC cells with DMSO or C646 treatment. (B) CoIP determined the interaction between P300 and CBP in LUSC cells. (C) qRT-PCR and western blot of P300 level under sh-P300 transfection in LUSC cells. (D) ChIP tested the H3K27ac in LINC00519 promoter in LUSC cells with P300 knockdown. (E) qRT-PCR of LINC00519 level under P300 knockdown in LUSC cells. (F) qRT-PCR and western blot of HDAC7 and PCAF levels under sh-HDAC7 or sh-PCAF transfection in LUSC cells. (G) Luciferase activity of LINC00519 promoter in LUSC cells with HDAC7 and PCAF knockdown. (H) qRT-PCR of LINC00519 level under in LUSC cells with HDAC7 and PCAF knockdown. ^**^P < 0.01. ^**^P < 0.01. n.s. indicated no significance.

**
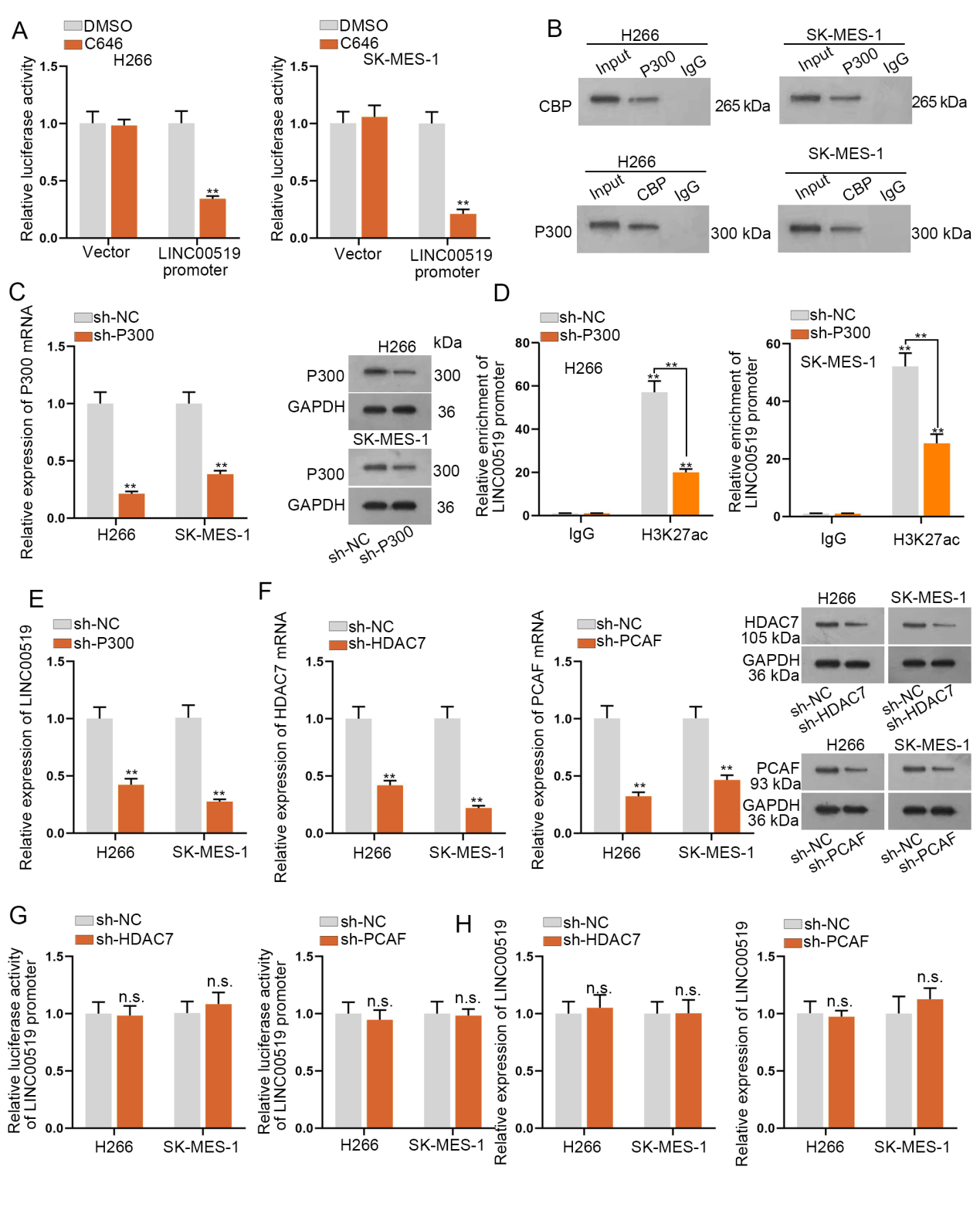
**

**Fig. S3 CBP/P300 regulated LINC00519 promoter H3K27ac and upregulation in LUAD cells.** (A) ChIP-qRT-PCR analysis of LINC00519 promoter enrichment in H3K27ac precipitates in LUAD cells versus normal HBE cells. (B) Western blot of P300 and CBP in LUAD cells with knockdown of P300 and CBP. (C) qRT-PCR of LINC00519 level in LUAD cells with knockdown of P300 and CBP. (D) ChIP-qRT-PCR analysis of LINC00519 promoter enrichment in H3K27ac precipitates in LUAD cells with knockdown of P300 and CBP. (E) Luciferase activity of LINC00519 promoter in LUAD cells with knockdown of P300 and CBP. ^**^P < 0.01.

**
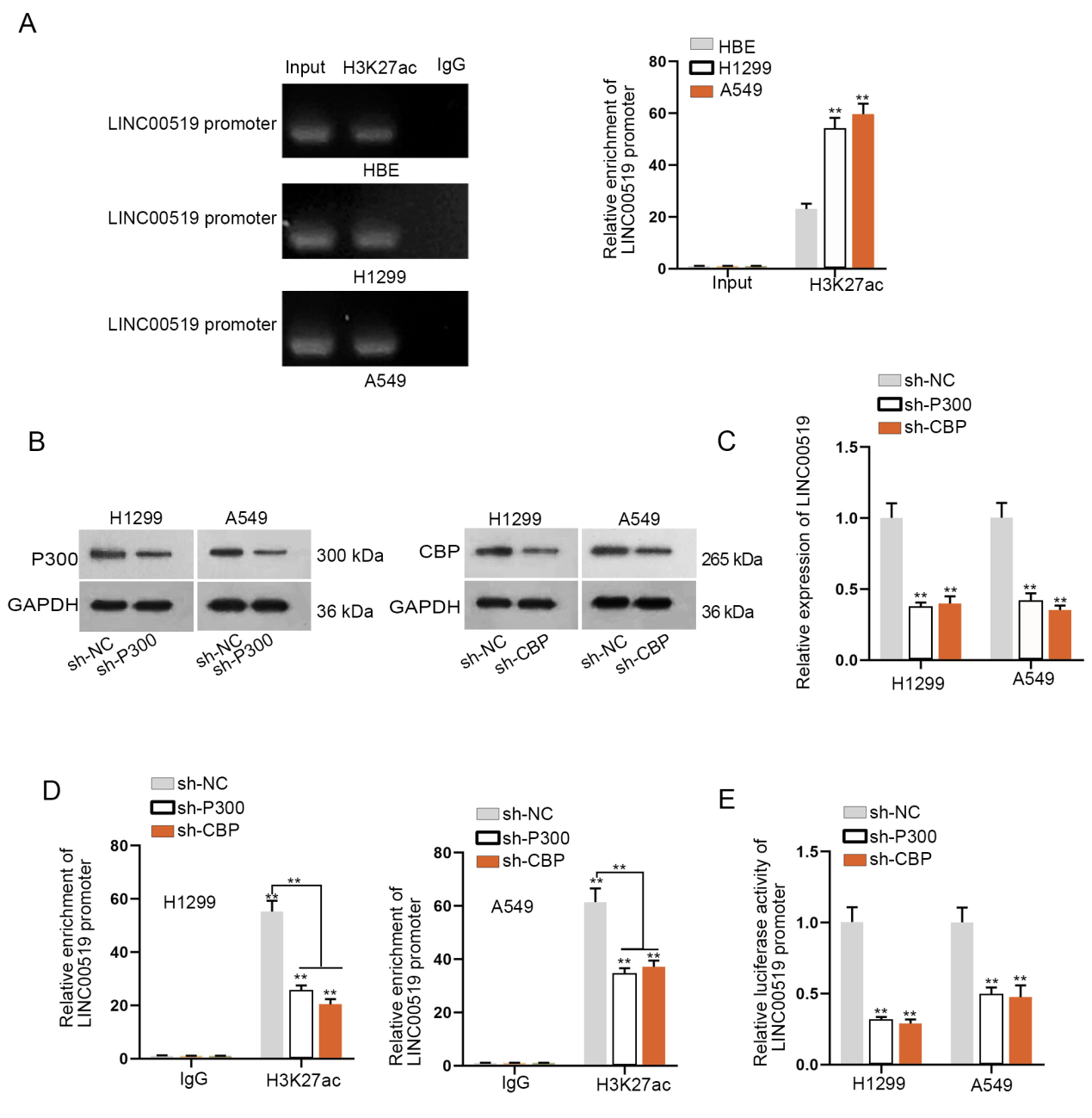
**

**Fig. S4 Confirmation of transfection efficiency.** (A) The expressions of miR-450b-5p and miR-515-5p were separately up-regulated in H266 and SK-MES-1 cells with the transfection of miR-450b-5p mimics and miR-515-5p mimics. (B) The overexpression vector was applied to up-regulate LINC00519 expression. (C) MiR-450b-5p or miR-515-5p expression was separately detected in miR-450b-5p mimics or miR-515-5p mimics transfected cells. (D) YAP1 expression was estimated in YAP1 overexpressed cells. ^**^P < 0.01.

**
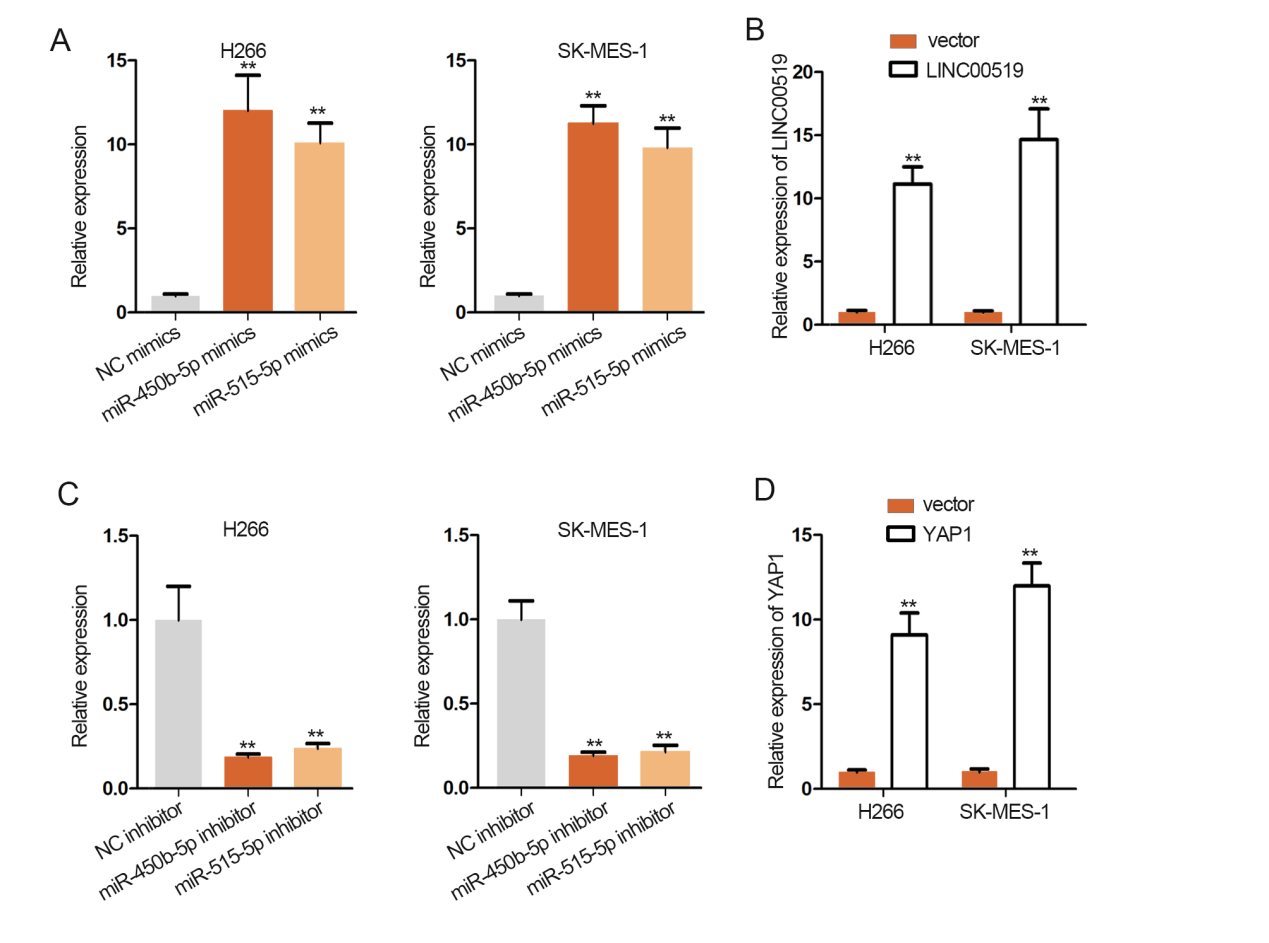
**

**Fig. 5 Original data of western blot.** (A-F) Original data of western blot for Figure 3F, 4B, 5J, S2C, S2F, and S3B.


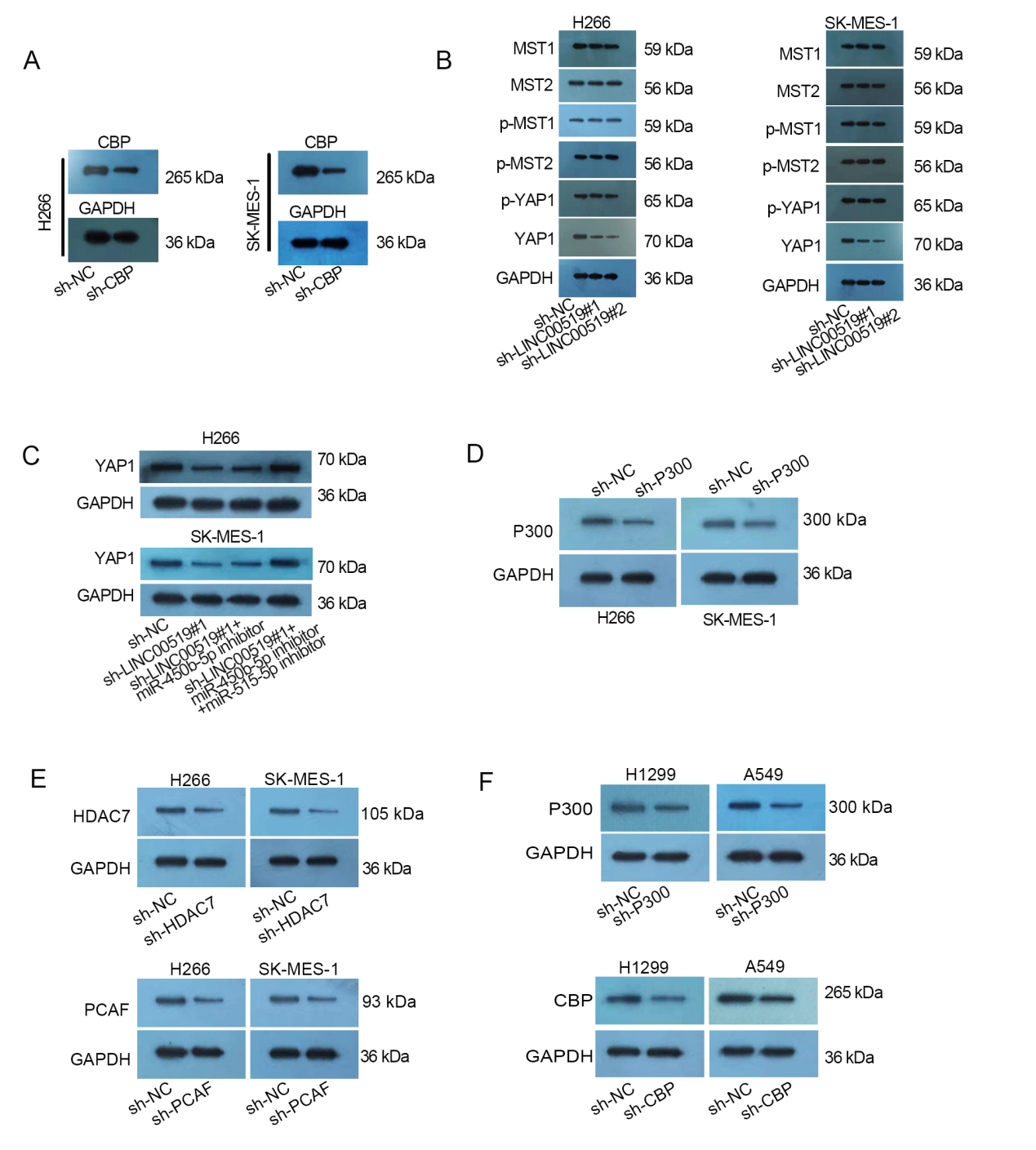

Supplement: Supplementary file 1 — Fig S1‐S5 [file CPR-53-e12797-s001.docx]
